# The burden of severe leukocyte adhesion deficiency, type I (LAD-I): A multiple-case study from the caregiver’s perspective

**DOI:** 10.70962/jhi.20260022

**Published:** 2026-06-04

**Authors:** Meaghan O’Connor, Clarissa Simas, Michael Keith, Miranda Bailey, Kristi Jackson, Leslie Blake, Maria Chitty Lopez

**Affiliations:** 1 IQVIA, Durham, NC, USA; 2 Rocket Pharmaceuticals, Cranbury, NJ, USA

## Abstract

Leukocyte adhesion deficiency, type I (LAD-I) is a rare inborn error of immunity characterized by recurrent, severe infections/inflammation and poor wound healing. Little is known about the patient/family experience of severe LAD-I. We conducted a multiple-case study exploring the burdens of illness, caregiving, and treatment in severe LAD-I. Nine caregivers (parents) representing five families with ≥1 child with severe LAD-I participated in individual, in-depth interviews. Memoing and inductive/deductive coding were used to analyze and compare each family’s experience. The caregivers described frequent and severe infections, physical and social restrictions, a complex journey to diagnosis, rearranging life around severe LAD-I, financial strain, isolation, and uncertainty. Treatment burden varied by treatment type. This multiple-case study highlights how severe LAD-I affects patients and families. As the first study to address the humanistic burden of LAD-I in patients and caregivers, it is an important contribution to the literature on LAD-I and qualitative methods for investigating the burden of rare disease on families.

## Teaser

This multiple-case study explores the burdens of illness, caregiving, and treatment for five families with children with severe leukocyte adhesion deficiency, type I (LAD-I). It highlights the experiences of nine caregivers and describes the ways their lives are impacted by this rare inborn error of immunity.

## Introduction

Leukocyte adhesion deficiency, type I (LAD-I) is a rare, autosomal-recessive inborn error of immunity (IEI) caused by mutations in the *ITGB2* gene (1). LAD-I typically presents with recurrent, often severe, bacterial and/or fungal infections affecting the mouth, skin, and respiratory tract; inflammatory complications; and impaired wound healing (2, 3). Umbilical complications (e.g., omphalitis, delayed separation of the umbilical cord) are a common first sign of the condition during infancy (4), while later, gingivitis and periodontitis are common hyper-inflammatory complications (2, 5). For children with LAD-I, infections often lead to hospitalizations and are the primary cause of morbidity and mortality (5, 6).

LAD-I is typically categorized as severe or moderate based on immunophenotype and/or clinical phenotype (7). Estimates suggest that one in 1 million individuals worldwide are affected by LAD-I (3, 8), and more than half present with severe LAD-I based on immunophenotype (5, 9). Following diagnosis, patients with LAD-I are typically started on prophylactic antimicrobials (e.g., antibiotics, antifungals) (5, 10). However, antimicrobials have not been shown to avert mortality in severe LAD-I. Indeed, studies have found that without allogenic hematopoietic stem cell transplant (allo-HSCT), only 31–39% of children with severe LAD-I will survive beyond age 2 (5, 11, 12). For the minority of children who do survive beyond age two without allo-HSCT, the risk of infection, infection-related complications, and death is known to increase or persist (7, 13, 14).

For >40 years, IEIs such as LAD-I have relied heavily on the development and refinement of allo-HSCT (15). However, allo-HSCT comes with risks, including transplant-related toxicities and graft-versus-host disease (16), which can render the transplant unsuccessful and/or further complicate the patient’s health (17). Additionally, finding a full-matched donor can be a lengthy and challenging process that is not always successful (13, 14). Gene therapies hold promise as a new, potentially curative option for IEIs (18, 19, 20), with recent evidence showing substantial clinical efficacy in some patients (13). During the period of this study, a lentiviral gene therapy for severe LAD-I (16) was undergoing clinical trial development (21, 22). This therapy has since received accelerated approval from the US Food and Drug Administration based on evidence indicating its potential as a definitive treatment option for this rare condition (18, 23, 24, 25).

Beyond the disease risks described above, research has demonstrated that living with a rare disease can significantly disrupt the lives of patients, families, and caregivers (26, 27). A 2021 systematic review of studies examining quality of life in parents of children with rare diseases found that, across most studies, parents of such children were more likely to report lower quality of life than parents of healthy children (28). We hypothesized that this would be the case for parents caring for a child with severe LAD-I, but, like many rare conditions, there is a paucity of published literature describing the lived experience of severe LAD-I. As such, we conducted a multiple-case study to better understand the humanistic burden (i.e., typical symptoms and impacts on daily life) of this rare condition.

## Results

### Case families

Five families participated in this study (Table 1). Four were in North America, and one was in Europe. All but one were represented by a mother and a father (family A was represented by the mother only) for a total of nine participants. Altogether, these families included seven children with severe LAD-I (ages 4–18 at the time of the interviews) and three children who did not have severe LAD-I. Among the children with severe LAD-I, four had received investigational gene therapy through an investigative clinical trial, two underwent allo-HSCT, and one was being treated solely with prophylactic antimicrobials at the time of interview.

**Table 1.** Case family characteristics

Parent/Study participant	Child	Diagnosed with severe LAD-I	Age at interview (years)	Treatment administered	
				Type	Age (years)
**Family A**	​	​	​	​	​
Amy (mother)	​	​	​	​	​
​	Allison (f)	Yes	14	Investigational gene therapy	8
​	​	​	​	​	​
**Family B**	​	​	​	​	​
Bridget (mother)	​	​	​	​	​
Bill (father)	​	​	​	​	​
​	Brooke (f)	Yes	18	Allo-HSCT	<1, 9
​	​	​	​	​	​
**Family C**	​	​	​	​	​
Christina (mother)	​	​	​	​	​
Chris (father)	​	​	​	​	​
​	Cara (f)	Yes	8	Investigational gene therapy	3
​	Caity (f)	Yes	7	Investigational gene therapy	2
​	Colby (m)	Yes	4.5	Investigational gene therapy	<1
​	​	​	​	​	​
**Family D**	​	​	​	​	​
Devi (mother)	​	​	​	​	​
Daniel (father)	​	​	​	​	​
​	Daisy (f)	No	12	n/a	n/a
​	Dev (m)	Yes	7	Allo-HSCT	<1
​	​	​	​	​	​
**Family E**	​	​	​	​	​
Eloise (mother)	​	​	​	​	​
Eric (father)	​	​	​	​	​
​	Emma (f)	No	10	n/a	n/a
​	Ellis (m)	No	6	n/a	n/a
​	Ethan (m)	Yes	3	Prophylactic antimicrobials	Ongoing

Allo-HSCT, allogenic hematopoietic stem cell transfer; f, female; LAD-I, leukocyte adhesion deficiency, type 1; m, male; n/a, not applicable. All names are pseudonyms to protect participant privacy and confidentiality.

**Figure 1.**
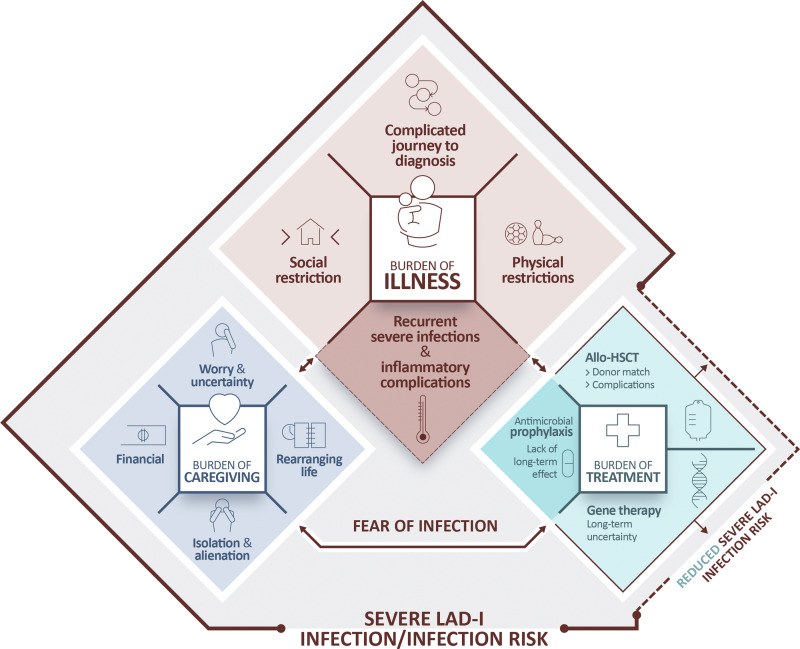
**Model demonstrating the experiences of six families experiencing severe LAD-I.** A graphical representation of the relationship between the burdens of illness, treatment, and caregiving experienced by families with children with severe LAD-I. The burden of illness is characterized by recurrent, severe infections and inflammatory complications, a complicated journey to diagnosis, and social and physical restrictions. The burden of caregiving is characterized by worry and uncertainty, financial strain, the need to rearrange life around the disease, and isolation and alienation. The burden of treatment is dependent upon the treatment type. Each burden exists within the context of the constant risk and fear of infection.

The three burdens (illness, caregiving, and treatment) are characterized by specific themes within them (Fig. 1); each theme is then described in detail below (and summarized in Table 2).

**Table 2.** Themes and examples of the burdens of illness, caregiving, and treatment in severe LAD-I

Burden	Themes	Examples
Illness	Frequent and varied infections	Rashes, skin/mucosal lesions, and infections in the lungs, ears, gums; poorly healing umbilical cords
	Physical restrictions	Avoiding water parks, beaches, other outdoor activities; limiting sports; preventing contact with pets
	Social restrictions	Limiting visitors to the home; avoiding daycare; homeschooling
	Journey to diagnosis marked by a lack of information	Failure of frequent severe infections and slow healing wounds to drive investigation and diagnosis; lack of resources, guidance, and information pre- and post-diagnosis
Caregiving	Rearranging life	Accommodating doctor and hospital visits, medication schedules; becoming a home-based educator; changing jobs, pausing, reducing, or rearranging (e.g., work from home, freelancing) work
	Financial strain	Lost wages and opportunities from declining or avoiding job advancements; lost wages from pauses in employment; costs of medical equipment, treatment, hospital stays, travel and accommodation associated with treatment; private insurance costs
	Isolation and alienation	Avoiding crowded spaces and events, family gatherings, and holidays; accessing nature in uncrowded space only; losing contact with friends and family; lack of connection with other families experiencing LAD-I
	Ever-present worry and uncertainty	Worry pre-diagnosis about the cause of illness, post-diagnosis about treatment efficacy, risks, and preventing infections; general hypervigilance; concerns regarding impact on the children (both those with and without LAD-I); uncertainty about their children’s futures
Treatment	Allo-HSCT	Searching for a bone marrow match; complexity of allo-HSCT process; managing allo-HSCT complications; side effects from post-transplant treatments; waning efficacy
	Investigational gene therapy	Generally positive outcomes, despite a long and complex process; required relocation for the duration of treatment; uncertainty about durability and longer-term effects given the novelty of gene therapy
	Prophylactic antimicrobials	In one case, in the absence of a family donor match for allo-HSCT, the clinician recommended this approach; parents indicated it is not a long-term or complete solution

### Burden of illness

Based on the descriptions of each parent’s observations of their child(ren)’s life with severe LAD-I, the burden of illness was characterized by frequent, often severe, infections and inflammatory complications; physical restrictions; social restrictions; and a journey to diagnosis that was marked by a lack of information.

#### Recurrent severe infections and inflammatory complications

The parents described their children’s slow-healing and infected umbilical cord stumps, rashes and skin infections, mouth sores, gingivitis, ear infections (leading to burst eardrums) and, for some, lung infections (Table 3).

**Table 3.** Severe LAD-I**–**related health issues

​	Allison	Brooke	Cara	Caity	Colby^a^	Dev	Ethan
Slow-healing, infected umbilical cord stump	​	​	•	​	​	•	•
Rashes	•	•	•	•	​	•	•
Skin infections	•	​	•	•	​	​	•
Mouth sores	​	​	​	•	​	​	​
Gingivitis	•	•	•	​	​	•	•
Ear infections	​	​	•	•	​	•	•
Lung infections (e.g., aspergillus)	•	•	​	​	​	​	​
Hospitalizations	•	•	•	•	​	•	•

a Infections/health issues were not described for Colby as he was diagnosed immediately after birth (given his siblings’ diagnoses) and received investigational gene therapy within months of birth.

Some of the infections experienced by the children were life-threatening and required intensive medical treatment: Allison was hospitalized with a severe lung infection, and as an infant, Caity was hospitalized after painful mouth sores kept her from eating. Caity’s mother, Christina (mother; family C), recalled:

*At that point, [Caity] had those mouth sores, and then she wouldn’t eat for... It was several days that she just wouldn’t eat anything… They had to admit her to the hospital…for, like, a week that time.*

#### Physical and social restrictions

To reduce the risk of infection, the children’s physical activities had to be limited to avoid injury and potential exposure to pathogens. Parents recalled their doctors cautioning against certain activities; for example, Amy (mother; family A), explained:

*[The doctor said that Allison] shouldn’t be around birds. She shouldn’t go to, you know, the public water parks… She shouldn’t dig in dirt. She shouldn’t garden… We couldn’t, we didn’t really go the beach… People that have LAD, they can’t be around all those things.*

Eloise (mother; family E) imposed limits on where and how her son, Ethan, could play, explaining:

*I prevent him from running on asphalt. You know, I wouldn’t allow him on a BMX [bike]. We practice hockey a lot…but I don’t let him play in his shorts only. I want him to wear protection… If he wants to go up a tree, it’s a “no.” If he wants to run on lots of rocks, it’s a “no.” You know? I am really trying to go where it's [safer], on the grass.*

The children’s social interactions had to be restricted to limit the risk of exposure to bacteria and fungi. These precautions limited participation in regular childhood experiences, such as playing outside, going to school, joining sports teams, and attending birthday parties. Even holidays and family gatherings were potential sources of infection, limiting interactions with extended family members. School and daycare attendance was also very complicated: catching a simple cold from a classmate could turn into a superimposed bacterial infection, leading to pneumonia, a hospital visit, and days or even weeks of missed attendance. As a result, five of the seven children in this study were homeschooled at some point. Brooke was one of the only children to attend school outside the home while dealing with severe LAD-I. However, that experience was fraught, as her mother, Bridget (mother; family B), recalled:

*If [Brooke] fell ill and had a cold, it would last. It wasn’t just a cold. Especially when she was younger, like, if she had a temperature, as soon as she hit 38° [Celsius; 100° Fahrenheit], we were in hospital anyway. So, she’d be on, you know, 2 weeks' antibiotics just in case it developed into something else… And then obviously, when—if she fell ill, she was out [of school].*

#### A journey to diagnosis marked by a lack of information

The journey to diagnosis for the participants’ children was complicated (Table 4): for some, it was long, and for all, it was marked by a notable lack of patient and family friendly information about severe LAD-I. Among the five families, the time-to-diagnosis varied from immediately following birth to 8 years from birth. Three of the children’s diagnoses were prompted by hospitalization for severe infections, and two were prompted by repeated poor wound healing or bleeding.

**Table 4.** Age and event prompting each child’s diagnosis of severe LAD-I

Child	Age at diagnosis	Event prompting diagnosis
Allison	8 years	Hospitalization for severe aspergillus infection in lungs
Brooke	2 wk	Hospitalization for an infected umbilical stump
Cara	3 years	Bleeding of the gums, which prompted the pediatric dentist to suggest seeking out an immunologist
Caity	2 years	Cara’s diagnosis prompted testing for both LAD-I and/or the ability to be an allo-HSCT MSD for Cara
Colby	Birth	Diagnosed at birth as a result of his older sisters’ diagnoses
Dev	6 mo	Hospitalization for a severe ear infection and burst eardrum
Ethan	3 years	Repeated experiences of poor wound healing

MSD, matched sibling donor.

For a few of the children, one of the first signs of severe LAD-I was omphalitis or delayed umbilical detachment. For Cara and Ethan, oral health issues (e.g., bleeding when brushing teeth, swollen gums) suggested to their parents and providers that something was seriously wrong. For example, Cara’s pediatric dentist noted her bleeding gums, felt they were likely due to an immunodeficiency rather than poor hygiene, and encouraged the family to visit an immunologist.

For most parents, the ability to obtain an immediate diagnosis of LAD-I for their child was delayed because doctors were unfamiliar with severe LAD-I and/or assumed the child was experiencing regular infant/childhood infections. For 8 years, Amy sought a diagnosis for her daughter. Throughout this period, doctors dismissed the rashes, coughs, and slow-to-heal wounds as normal childhood illnesses.

Although a diagnosis did provide the parents with some relief, they nevertheless found that the frustration and uncertainty continued. Each of the parents remarked on the lack of available information about severe LAD-I, particularly patient and family friendly information. While their children’s clinicians provided some information, parents were eager to understand more and sought details on the internet, encountering information that was either too difficult to understand (e.g., too technical) or, simply, too terrifying (e.g., the high mortality rates).

Three of the five families (families A, D, and E) noted that doctors’ lack of awareness of the condition continued to be an issue after diagnosis. These parents recalled seeing clinicians in primary care or general hospital settings who were unfamiliar with LAD-I. Amy explained that her daughter’s primary care doctors lacked resources and, “Because this is such a rare disease, as we were searching for answers, even the doctors were guessing.” For Eloise, the lack of familiarity with severe LAD-I among healthcare providers at the local hospital caused significant stress and worry: once, when her son Ethan was hospitalized for an infection, he was placed in a room with other sick children. This was distressing for her, knowing how easily he could pick up yet another infection. She knew she could provide valuable information to the clinicians who admittedly did not know what to do, but despite making a plea for a private room, her request was dismissed.

### Burden of caregiving

The challenges faced by children with severe LAD-I directly impacted the burden their parents faced as caregivers (particularly prior to potentially definitive treatment). The parents rearranged their lives around severe LAD-I and experienced financial strain, isolation and alienation, and worry and uncertainty.

#### Rearranging life

Caring for a child with severe LAD-I required orienting life around avoiding infection, caring for illness, and frequent doctor’s office and hospital visits. Because their children were unable to go to daycare or school, each of the mothers also became their child(ren)’s educator. Amy and Christina both described how they converted space in their homes into a classroom to homeschool their children. Each of the mothers stopped working outside the home or altogether for a period to provide full-time care and education to their children at home, where infection risk could be better controlled. Daniel (father; family D) recalled the time during which his wife, Devi (mother; family D), stayed home full-time with their son:

*[Dev’s] health was so fragile that he had to be admitted, inpatient, into [major hospital]. I think he was in there for six months or something like that. [Devi] was there Monday through Friday; she stopped working, and she was there, Monday through Friday, in hospital.*

Each of the fathers also described ways in which their career choices, and possibly their career trajectories, were impacted by their children’s severe LAD-I. Daniel reflected on how his decision to remain in his current job, coupled with his wife’s decision to stop working to care for Dev full-time, likely impacted both of their career trajectories:

*[Devi] had this huge gap in her resume for, like, “X” amount of years. I would like to think that if I had not been in the defensive crouch, I would have been trying to seek some kind of additional career advancement or whatever. But I had a good thing going with the health insurance. Trying to start a new job at some place with a child with, you know, extraordinary demands seemed to be a big unknown. That [was] a risk that I was…not willing to take.*

#### Financial strain

Caring for a child with severe LAD-I caused financial strain in some capacity for each of the families. For some, it was driven by the need to pause work or pass on potential career advances. For others, financial strain was driven by the cost of care itself, including frequent doctor visits, hospital stays, and costly treatments. Whether the cost of care was an issue depended on the healthcare system in which each family was operating. Those with public insurance (families A, B, and E) experienced few to no healthcare-related expenses. Those families that relied solely on private healthcare insurance (families C and D), however, experienced significant financial strain due to medical and treatment costs. Christina and Daniel both described the high costs of care that they had to contend with during their children’s severe LAD-I treatment:

*Every year [prior to gene therapy], we met our out-of-pocket [maximum] for insurance, which is... it’s crazy! And it’s taken us really until this last year [5 years after treatment] to, to get ahead of everything and get back on our feet and get to a better place financially. *(Christina; mother, family C)

*I think [his care] soaked up three quarters of a million dollars, and we didn’t care. [We had] a lot of medical expenses, a lot of caregiving expenses, a lot of expenses for trying to retrofit our lives to minimize viral exposure. *(Daniel; father, family D)

#### Isolation and alienation

When managing severe LAD-I (specifically prior to allo-HCST or gene therapy), each of the families was extremely cautious, often to the point of isolating themselves both physically and socially to avoid the risk of infection. Christina recalled, “When you’re just trying to protect your kids…you do not feel like you can leave the house at all. I mean, I wouldn’t take them to the grocery store even after [their diagnoses].” Each of the parents described avoiding crowded spaces or events. Even gatherings with family and/or friends could pose a risk:

*Even people around us, as they learned that [Allison] had, you know, a deficiency…if we were supposed to have like a school event, they would tell me, “Oh, you might not want to come…because so and so’s family has been sick,” or whatever. *(Amy; mother, family A)

Bill and Bridget (father and mother, respectively; family B) described vacations to open spaces, which felt safer to them because they were less likely to encounter crowds and, thus, fewer opportunities to contract an infection. As Bill explained, “Well, we’ve always gone on holiday and…we kind of get our own space, away from busy places.”

The physical isolation families enacted to keep their children safe and healthy frequently turned into social isolation as well, leading parents to feel alienated from others. For example, Bridget explained:

*It affects your relationships with other people…My dad was poorly when [Brooke] was…only 2. And I felt like I couldn’t…I wasn’t there to see him because he was in hospital; I was scared of picking something up [getting sick]. And he was telling me, “I don’t want you coming into hospital ’cause I do not want you getting anything from here.” It just affects everything. It affects your whole family.*

The lack of greater awareness about severe LAD-I, paired with the gravity and struggles of the condition, made it difficult for parents to relate to others, including friends and family. As severe LAD-I (and their child[ren]’s survival) became their sole focus, the parents found themselves withdrawing from relationships. Daniel recalled that he and his wife “would try to socialize, try to…reconnect with old friends and stuff” but found that it could “be very alienating” because “your options are either to trauma-dump on them…and I realized that, at a point, as concerned as people are, nobody wants to hear, like, all your [stuff].”

The isolation and alienation were compounded by the lack of access to other families/parents with children with severe LAD-I. Most parents expressed a desire to connect with other families and parents of children with severe LAD-I, as it was unlike other childhood or chronic diseases. Amy explained that she would have liked “a support system of people who understand what you’re going through. Specifically, a network of other parents of children with LAD-I… It can be really lonely without that.” Eloise echoed this sentiment:

*I think I looked at all the Facebook pages for rare diseases, in order to find someone to talk to. You know someone with cancer; we know many of them, [so], you know, we can talk about [that]. But not about LAD-I.****[What would you like to share with them?]****[Sighs] Simply having someone who understands me.*

#### Worry and uncertainty

The constant worry and fear that accompanied caring for a child with severe LAD-I was a shared experience across the parents. Before their children’s diagnoses, parents worried why treatments were never fully effective and why serious and severe infections continued to occur. Once severe LAD-I was confirmed, new worries regarding the poor prognosis of the condition were coupled with fear for their child’s future. Devi recalled the fear she experienced when Dev was first diagnosed and they were sent home from the hospital while they waited for more information on allo-HSCT:

*I was at home, and I was like…“Should I be wiping myself with alcohol swabs to nurse him? What should I be doing?” And so, I kept on calling [the doctors]. And then they would be like, “You don’t need to be so nervous, like, stop panicking…” [But] I am here trying to figure out how to keep him alive until we can get him to transplant.*

Each parent described an almost constant and overwhelming worry about their children’s vulnerability to infection during active LAD-I. Eloise described worrying as she watched her son, Ethan, play, knowing that even the smallest scrape might lead to a potentially life-threatening infection, while Bill described his family’s experience as “18 years of...24/7 worry.”

### Burden of treatment

The burden of treatment varied according to treatment. Upon diagnosis, all five families were told by their doctors that the treatment for severe LAD-I was allo-HSCT (communicated using the term “bone marrow transplant”). Four of the families pursued finding an allo-HSCT match for their children. Family C went through a long, unsuccessful process trying to find matches for their children. Each of the members of family E were tested, but the results showed that none were a match for Ethan. Daniel recalled that “it took a long time, unfortunately…for them to scour the worldwide registry for marrow donors” for Dev. A perfect match was not available for Dev, nor for Brooke, resulting in the use of close matches for each of them.

Of the two children that had allo-HSCT, results were mixed. While Dev’s transplant effectively treated the severe LAD-I, a host of other issues arose due to the transplant itself (e.g., graft-versus-host complications, the need for long courses of steroids, continued vigilance around the risk of infection). The road to recovery for Dev was more than a year long, but despite these challenges, the transplant was a success. His health improved markedly over time, and, in turn, Devi was able to return to work full-time. In contrast, Brooke had two transplants. The first, during infancy, was unsuccessful, and the second, 8 years later, was followed by a challenging recovery due to graft-versus-host disease. Nevertheless, the second transplant did reduce her risk of infection and give her the opportunity to attend school outside the home and spend time socializing friends more freely. The reduction in infection risk afforded by the transplant also meant that Bridget could return to full-time work outside of the home. Although years had gone by since the second transplant, Brooke’s parents expressed concern about indications that her second transplant may also fail. As such, the future remained uncertain for this family.

In contrast, after investigational gene therapy, the children in families A and C were no longer experiencing frequent, serious infections, and doctor visits were limited to well-child checkups. Amy recalled that prior to gene therapy, Allison “was on 8, 9, 10 medications during the day…and now, she’s just on multivitamins and, like, probiotics: you know, just the normal stuff that kids take.” No longer requiring physical and social restrictions to avoid the risk of infection meant these children were able to participate in social activities and attend school outside the home. Both Amy and Christina were able to return to work as well. Despite the positive outcomes, the process to receive gene therapy was long and complex, requiring lengthy hospital stays and significant travel for both families. Family A traveled to and remained at the clinical trial site for 2 mo, while family C moved to the clinical trial site for 6 mo. To accommodate these long stays, Amy took a break from her graduate school program, while Chris switched from an in-person position with his company to a fully remote position. Finally, Christina and Chris, while grateful for the positive outcomes of investigational gene therapy, did describe some concerns about the durability of the treatment’s effects along with uncertainty regarding unknown or unexpected effects that might manifest in the future. Christina explained:

*We don’t know what the future holds since [the gene therapy] was a clinical trial. It is experimental. There are risks that come with that and so, you know, potential for things to show up later in life that we just don’t know about…which was a risk we felt like we had to take [at] the time to get the time with them now.*

Prior to potentially definitive treatment, all of the children described in this study used antimicrobials (antibiotics and/or antifungals), either prophylactically or in response to acute infections associated with severe LAD-I. The parents all agreed that antimicrobials alone were insufficient in the long term for their children. And while Ethan was able to live a relatively unrestricted life (i.e., a life without constant, severe infections and hospitalizations) using only prophylactic and responsive antimicrobials, Eloise acknowledged that allo-HSCT was likely in his future.

### Disease journey by treatment type

**Figure 2.**
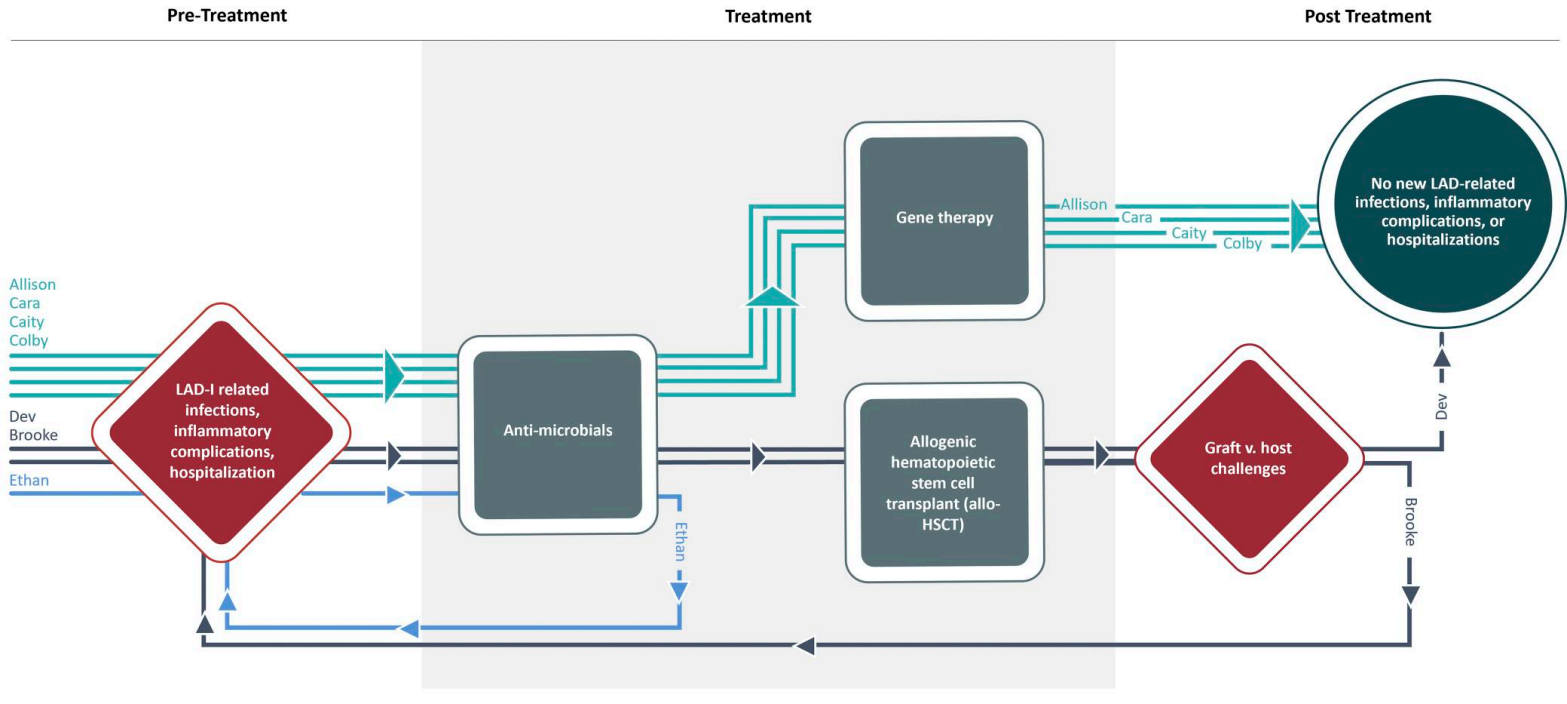
**Participants’ children’s severe LAD-I disease journeys by treatment type.** A graphical representation of the different disease journeys experienced by each of the children with severe LAD-I whose parents participated in the study. Each child started with the shared experience of severe, recurrent infections, inflammatory complications, and hospitalizations and was initially treated with antimicrobials. The journeys then diverge. Children who had gene therapy no longer experienced LAD-related infections, etc. Those who had allo-HSCT experienced graft-versus-host challenges and either stopped experiences infections or needed a second stem cell transfer. The child who was only treated using antimicrobials continued to be in a loop of infection/inflammation and antimicrobial treatment.

Although recurrent health issues driven by infection are central to the severe LAD-I experience, the experiences of the families in this study demonstrate how the trajectory of those infections can change significantly depending on treatment (Fig. 2).

Children in families A (Allison) and C (Cara, Caity, and Colby) who had investigational gene therapy experienced the most significant improvements; after treatment, they were rarely sick, went to the doctor only for well-child visits, and could play, go to school, and interact with others without fear of infection. The children in families B (Brooke) and D (Dev) who had allo-HSCT faced more challenging recoveries and different trajectories toward good health. While Dev’s transplant was ultimately successful, Brooke’s first transplant was unsuccessful, sending her back into the experience of recurrent infections. Her second transplant was partially successful, allowing her to attend school with others, go out with friends, and travel; however, infections were still an issue for her, and as the second transplant’s effectiveness waned, infections continued to be a concern. Lastly, Ethan, whose only treatment was prophylactic antimicrobials, remained in a loop of infections and treatment.

## Discussion

The results of this multiple-case study offer valuable insights into life with severe LAD-I. Each of the case families was unique in its makeup and location, but the similarities across their experiences of severe LAD-I resulted in many commonalities. The connection between severe, life-threatening infections and inflammatory complications and patients with severe LAD-I is well-established: the omphalitis, poor wound healing, skin lesions, and gingivitis experienced by the children in this multiple-case study were consistent with the five children with LAD-I described by Gorjipour et al. (2019) in their case series. However, while Gorjipour et al. limit their descriptions to the more clinical aspects of their cases (29), this study goes further by delving into the impacts on children’s lives. It highlights how, to reduce infection risk, the children were isolated physically and socially, limiting their ability to attend school, play with friends, and explore the world around them.

The burden faced by the caregivers in this multiple-case study also mirrors that observed in other conditions (30). The parents in this study were wholly oriented around their child’s condition, often to the detriment of their own wellbeing as they became more isolated (physically, socially, and emotionally) to reduce their child(ren)’s risk of a life-threatening infection. These experiences aligned with those identified in a systematic review by Liu, Heffernan, and Tan (2020) (30), in which the authors noted that across the studies they examined, caregivers experienced a multifaceted strain defined by health problems, emotional and psychological distress, alienation or deterioration of family relationships, and disruptions to their schedule and lifestyle that ultimately led to economic strain, reduced socialization, and isolation (30). This same multifaceted strain is demonstrated in the experiences of the parents in this study who described rearranging their lives around severe LAD-I, assuming additional roles and responsibilities, feeling isolation and alienation, financial challenges, and navigating life and raising children against a backdrop of constant worry and uncertainty. These experiences are also consistent with the challenges faced by patients and caregivers in other rare diseases, such as complex (and often delayed) journeys to diagnosis (31, 32) and relocating to be closer to critical care resources (32), as well as a lack of disease-specific information and support groups (33).

In terms of the burden of treatment of severe LAD-I, the cases profiled here highlight some of the risks and benefits of the different treatment options, as experienced by these families. While prophylactic and reactive antimicrobials may have been a short-term solution for these children (and one that comes with its own risks, such as the possible development of antibiotic resistance), effectively addressing the infections and inflammation of severe LAD-I long-term required something more. Early action with allo-HSCT or, if available, a clinical trial of gene therapy, allowed most of the children to reduce or eliminate the symptoms and impacts of severe LAD-I. It also eliminated the need for physical and social isolation, allowing the children and their families to shift their focus from surviving to thriving.

The cases described in this study highlight areas where more can, and should, be done to improve the experience and burden of severe LAD-I. First, the families in this study wanted patient friendly information about the condition and opportunities to connect with one another. An LAD-I-specific patient/caregiver network has the potential to make a positive impact on the lives of families grappling with severe LAD-I, helping to increase knowledge and decrease isolation. Second, the families in this study grappled with clinicians who were unfamiliar with severe LAD-I. Better education for all clinicians, not just specialists, regarding signs, symptoms, and risks may help children to receive a diagnosis, start on appropriate treatment, and chart a path forward more quickly, a goal that is particularly important given the high risk of mortality for children with severe LAD-I.

This study was not without limitations. For participants whose children underwent successful treatment, descriptions of life with active infections and inflammation due to severe LAD-I were retrospective and therefore potentially subject to recall bias. However, that each caregiver was able to describe, in depth, the various hospitalizations, tests, and limitations that their child(ren) had to manage and the steps they took to accommodate the condition in their lives suggested that none struggled to recall life before or during treatment, and thus, recall bias likely did not affect their responses. Additionally, as a multiple-case study with a small group of participants and unequal representation among the treatment types, the results may not be generalizable beyond the families profiled here. Further, as gene therapy is a fairly recent innovation in severe LAD-I, it is possible that the experiences of caregivers and their children may vary more as more time passes posttreatment. Despite this, the results nevertheless make a useful contribution to the literature, given the lack of published information describing the caregiver experience of severe LAD-I, and can point researchers toward concepts that may be important for further exploration in larger sample sizes (where possible), different populations (e.g., patients, siblings), and/or in future single- or multiple-case studies.

## Materials and methods

Given the rarity of LAD-I (and thus, recruitment challenges) and the unique experience of the condition, we selected a multiple-case study design for our study as case studies are a valuable method for examining a distinct phenomenon in depth and in a specific context (34). Further, a multiple-case study design can explore how two or more similar situations may produce the same or different results as each case is analyzed individually and in comparison (34).

The study protocol was reviewed by WCG IRB and granted exemption. Nevertheless, all participants were provided with informed consent documentation prior to data collection, and each participant provided verbal consent before interviews commenced.

Leveraging existing relationships with clinicians, we used purposive sampling to identify families with children with severe LAD-I located in North America and Europe. We aimed to enroll two caregivers from each family with the goal of conducting at least one interview per caregiver. We also aimed to include a mix of severe LAD-I treatment experiences among the families to allow for comparison across treatment modality. Six families were considered for inclusion in the study. To limit the number of study team members with access to participants’ identifying information, communication with potential participants was limited to the study’s principal investigator (M. O’Connor), a trained qualitative researcher.

Data collection took place from April 2024–June 2024. Interviews were conducted in English (60 min) or French (90 min, to accommodate translation), depending on the caregiver’s preferred language. All interviews followed the same semi-structured guide. One participant who was the sole representative of their family also participated in a follow-up interview that further explored the topics discussed in their first interview. Interviews were conducted by a single researcher (M. O’Connor), with support from a skilled French-English interpreter (trained on the study objectives and interview guide) when relevant.

Interviews were conducted in-person at quiet, private locations convenient for the participants. Interviews for each family took place either consecutively on a single day or over 2 days. Interviews were audio-recorded (with the participant’s permission) and later transcribed verbatim, with all identifiable information removed. Each family was assigned a unique study letter, and participants and their children were each assigned a corresponding pseudonym beginning with this letter to maintain participant confidentiality and privacy.

After each set of interviews (i.e., two interviews per family), the study team discussed emerging themes, similarities or differences between families, and topics for exploration in subsequent interviews. The study team relied on memoing (i.e., writing reflections and insights immediately following the interviews and team discussions), an approach that allows researchers to conceptualize raw data in a way that explains a phenomenon in its context (35). Traditional inductive and deductive coding were also used. Memoing and coding were completed by M. O’Connor and C. Simas. The final analysis was generated by M. O’Connor, K. Jackson, and C. Simas through a collaborative approach. Analysis focused on describing and comparing each family’s experience in the context of the three burdens: illness, caregiving, and treatment, each of which was defined based on existing literature (30, 36, 37).

### Conclusion

This multiple-case study illuminates the experiences of both children coping with severe LAD-I and the parents who care for them. In so doing, it shows how disruptive this disease can be as infection control and risk reduction become a family’s sole focus. The burdens faced by these families were all-encompassing and, in many ways, mutually reinforcing, raising and/or exacerbating one another, resulting in a complex and often lonely experience. This multiple-case study marks an important step toward both understanding and sharing the experience of severe LAD-I from the perspective of families who have lived with it and making recommendations for concrete steps that can potentially reduce the burden on families. This study also demonstrates the effectiveness of the multiple-case study approach to elucidate the caregiver experience of a rare disease by focusing on depth instead of breadth. In so doing, it can serve as an example of the methodology that can be adapted to other rare diseases and participant groups, including but not limited to caregivers and patients.

## Ethics approval and consent to participate

This study was reviewed and granted exemption by WCG IRB. All participants received a study information sheet with details about the study prior to being interviewed. Participants also provided verbal consent at the start of each interview.

## Data Availability

The data underlying this study are not publicly available to maintain participant privacy.
